# Supplementary material for: An evaluation of the evidence submitted to Australian alcohol advertising policy consultations
Source: PLoS One. 2021 Dec 10;16(12):e0261280. doi: 10.1371/journal.pone.0261280 (PMC8664180; doi:10.1371/journal.pone.0261280)
Supplement: S1 Table — (DOCX) [file pone.0261280.s001.docx]

S1 Table. Number of submissions by actor type

|  | Industry actors | | | | | Non-industry actors | | | | | |  |
| --- | --- | --- | --- | --- | --- | --- | --- | --- | --- | --- | --- | --- |
|  | Advertising and media organisations  n (%) | Alcohol trade associations  n (%) | Alcohol producers or retailers  n (%) | Sporting organisations  n (%) | Industry actor  total  n (%) | Non-government organisations  n (%) | Medical colleges / health providers  n (%) | University / research institutes  n (%) | Government agencies  n (%) | Individuals  n (%) | Non-industry actor total  n (%) | Total of all submissions  n (%) |
| Submissions to ANPHA consultation | 11 (37) | 5 (17) | 4 (13) | 0 (0) | 20 (67) | 6 (20) | 0 (0) | 1 (3) | 2 (7) | 1 (3) | 10 (33) | 30 (100) |
| Submissions to NSW Bill consultation | 12 (29) | 7 (17) | 4 (10) | 2 (5) | 25 (61) | 6 (15) | 5 (12) | 4 (10) | 1 (2) | 0 (0) | 16 (39) | 41 (100) |
| Total | 23 (32) | 12 (17) | 8 (11) | 2 (3) | 45 (63) | 12 (17) | 5 (7) | 5 (7) | 3 (4) | 1 (1) | 26 (37) | 71 (100) |

Note. ANPHA: Australian National Preventive Health Agency issues paper (Alcohol advertising: The effectiveness of current regulatory codes in addressing community concerns). NSW: New South Wales Alcoholic Beverages Advertising Prohibition Bill.
