# Supplementary material for: An evaluation of the evidence submitted to Australian alcohol advertising policy consultations
Source: PLoS One. 2021 Dec 10;16(12):e0261280. doi: 10.1371/journal.pone.0261280 (PMC8664180; doi:10.1371/journal.pone.0261280)
Supplement: S2 Table — (DOCX) [file pone.0261280.s002.docx]

S2 Table. Accessibility of cited evidence by actor group, consultation and publication type

|  | Cited evidence in industry actor submissions | | | | Cited evidence in non-industry actor submissions | | | |
| --- | --- | --- | --- | --- | --- | --- | --- | --- |
|  | ANPHA  n accessible (n not accessible) | NSW  n accessible (n not accessible) | Total  n accessible (n not accessible) | Proportion of cited evidence not accessible  % | ANPHA  n accessible (n not accessible) | NSW  n accessible (n not accessible) | Total  n accessible (n not accessible) | Proportion of cited evidence not accessible  % |
| Peer-reviewed journal | 46 (0) | 119 (0) | 165 (0) | 0 | 115 (0) | 161 (0) | 276 (0) | 0 |
| Academic publication | 14 (7) | 8 (0) | 22 (7) | 24 | 29 (7) | 16 (0) | 45 (7) | 13 |
| Government publication | 45 (5) | 29 (6) | 74 (11) | 13 | 53 (11) | 63 (4) | 116 (15) | 11 |
| Parliamentary publication | 6 (0) | 4 (1) | 10 (1) | 9 | 7 (2) | 6 (0) | 13 (2) | 13 |
| Publication by an alcohol industry-linked organisation | 20 (22) | 19 (15) | 39 (37) | 49 | 10 (5) | 6 (0) | 16 (5) | 24 |
| Publication by an organisation not linked to the alcohol industry | 22 (1) | 15 (1) | 37 (2) | 5 | 59 (4) | 62 (1) | 121 (5) | 4 |
| Publication from an alcohol advertising self-regulatory body | 64 (2) | 30 (6) | 94 (8) | 8 | 65 (1) | 18 (1) | 83 (2) | 2 |
| Media coverage | 1 (0) | 1 (0) | 2 (0) | 0 | 8 (4) | 8 (0) | 16 (4) | 20 |
| Unknown publication type | 2 (4) | 2 (3) | 4 (7) | 64 | 0 (4) | 0 (0) | 0 (4) | 100 |
| Total accessible (not accessible) | 220 (41) | 227 (32) | 447 (73) | 14 | 346 (38) | 340 (6) | 686 (44) | 6 |

Note. Peer-reviewed journal papers were categorised as accessible if at least the abstract was accessible. Cited evidence that was not accessible either i) had an incomplete bibliography entry that did not include enough detail to locate, ii) could not be located from a Google search, iii) was a webpage where the content had likely changed or was no longer accessible, iv) was from a fee-payable source (e.g., OzTAM, Nielsen), v) was a book that was not available at the university library or online, or vi) was referred to as “unpublished”, “private report”, or “personal communication” in the citation or bibliographic entry. ANPHA: Australian National Preventive Health Agency issues paper (Alcohol advertising: The effectiveness of current regulatory codes in addressing community concerns). NSW: New South Wales Alcoholic Beverages Advertising Prohibition Bill.
