# Supplementary material for: An evaluation of the evidence submitted to Australian alcohol advertising policy consultations
Source: PLoS One. 2021 Dec 10;16(12):e0261280. doi: 10.1371/journal.pone.0261280 (PMC8664180; doi:10.1371/journal.pone.0261280)
Supplement: Attachment — Submitted filename: Response to Reviewers.docx [file pone.0261280.s003.docx]

| **Comment** | **Response** |
| --- | --- |
| **Editor** |  |
| 1. I also concur with Reviewer 1 that the material under Acknowledgments is more appropriately located within a competing interests statement - I ask that you review the journal's policy on declaring competing interests to decide what is most appropriately declared. I do not necessarily think this needs to be included in the limitations section, but you might include a paragraph on 'reflexivity' if you think it important to document how the team's expertise and social location shaped the work and in what ways. I look forward to receiving your revised manuscript. | Thank you for your guidance on the appropriate placement of our disclosures regarding contributions to submissions and the drafting of the ANPHA final report.  We have reviewed the competing interests policy that applies to PLOS journals. The contributions of author/s to the submission to the ANPHA issues paper and the ANPHA expert committee occurred more than five years before the research under consideration was conducted; however, we have disclosed these contributions in our competing interests statement to maximise transparency.  We have revised our competing interests statement by adding the following text:  *I have read the journal’s policy and the authors of this manuscript have the following competing interests: JS and HP contributed to the written submission of the McCusker Centre for Action on Alcohol and Youth to the inquiry into the NSW Alcoholic Beverages Advertising Prohibition Bill 2015 and JS appeared as a witness to a public hearing held for the inquiry. JS and HP contributed to the written submission of the McCusker Centre for Action on Alcohol and Youth to the ANPHA issues paper. TC was a member of the Australian National Preventive Health Agency’s expert committee on alcohol and contributed to drafting of the final report.*  All of the ‘Acknowledgements’ content of the original manuscript is now included in the competing interests statement and we have removed the ‘Acknowledgements’ section of the revised manuscript. |
| 2. Please amend your current ethics statement to address the following concerns:  a) Did participants provide their written or verbal informed consent to participate in this study?  b) If consent was verbal, please explain i) why written consent was not obtained, ii) how you documented participant consent, and iii) whether the ethics committees/IRB approved this consent procedure. | The present study involved analyses of documents only and did not involve human participants. The study forms part of the first author’s PhD research with multiple studies. Ethics approval was obtained for all components of the PhD research together. As the present study does not require an ethics statement according to the journal’s guidance, we have indicated N/A in the ethics statement field and removed the sentence from the methods section that indicated ethics approval. |
| 3. Please ensure that your manuscript meets PLOS ONE's style requirements, including those for file naming. | We have uploaded two copies of the manuscript labelled ‘Revised Manuscript with Track Changes’ and ‘Manuscript’. |
| 4. Please review your reference list to ensure that it is complete and correct. | We believe the reference list is complete and correct. We have added four references to the reference list of the revised manuscript (see points 17 and 20). |
| **Reviewer 1** |  |
| 5. This is a very well designed, explained and executed piece of research that will no doubt contribute beneficially and insightfully to the field. The research has been rigorous and is well contextualised and explained in relation to relevant literature, in particular on alcohol and secondarily, tobacco. Quite often, questions that did arise were subsequently addressed in the later discussion. The findings have yielded significant descriptive statistics that help substantiate the conclusions. The comments below are minor and there were no issues found of any substance that would undermine a very positive evaluation of the standard and significance of the research. The authors are to be commended. | We appreciate this positive feedback. |
| 6. The Abstract is very good and gives a succinct summary of the research. I would recommend adding to the introductory Abstract paragraph ‘Evaluation of “The value of the evidentiary contribution of industry actors to consultations on alcohol advertising policy appears to be limited” | Thank you for this suggestion. We have amended line 50 (p.3):  *Evaluation of* the value of the evidentiary contribution of industry actors to consultations on alcohol advertising policy appears to be limited. |
| 7. Body of the article p.8 of the MS pdf. Re: ‘ls of cited evidence were extracted and coded against a framework adapted from previous research (primary codes: subject matter relevance, type of publication, time since publication, and independence from industry)’. Q might the authors consider weighting the criteria with independence from industry the most highly weighted? | As this was the first time (to our knowledge) that evidence in submissions about alcohol policy had been assessed for relevance and quality, we treated the criteria equally. Our approach was consistent with Hatchard et al.’s (2014) analysis of evidence cited in tobacco policy submissions.  We have added a sentence to the discussion section ‘Unanswered questions and future research’ (lines 477-479, p.25):  *Future assessments of the relevance and quality of evidence may consider weighting the criteria to prioritise the indicators of greatest importance to the issue under consideration.*  We agree with Reviewer 1 that independence from industry is an important indicator of quality. As per the study’s limitations (p.24-25), it was disappointing that almost one-third of journal and academic publications cited in submissions did not include a statement from which independence could reasonably be assessed. |
| 8. p. 12 of the MS pdf. Line 115-16 Should say investigations by researchers [ie in order to differentiate this from governments] | Thank you for this suggestion. We have amended line 116 (p.6):  Investigations *by researchers* of alcohol industry efforts to influence advertising policy… |
| 9. method p. 13 of the MS pdf. Q why would human subject ethics permission be sought to examine publicly available submissions? Perhaps other aspects of the research should be mentioned in this regard eg. Interviews or whatever necessitated human subject research. | There are no other relevant aspects of the research to be mentioned in the manuscript and we apologise for the confusion. As noted at point 2 above, we have indicated N/A in the ethics statement field and removed the sentence from the methods section that indicated ethics approval. |
| 10. p. 13 of the MS pdf. the two inquiries that constitute the source of data are well described. However the authors could say why it was important. Eg was it to compare the two as justification that the results were not ideosyncratic to one inquiry? Was it to include both national and state inquiries etc.? | Thank you for this suggestion. We sought to include all the public consultations (state/territory and federal level) from the past 10 years that primarily focused on alcohol advertising policy to increase the likelihood that our findings would be relevant beyond a single consultation.    We have added a sentence to the ‘Strengths and limitations of the study’ section of the discussion (lines 453-454, p.25):  *The inclusion of multiple consultations strengthened our ability to consider the implications of findings for improving the use of evidence in policy processes.* |
| 11. p.16 of the MS pdf. Most of the codings are well explained and where relevant, the source in other relevant research. Regarding time since publication defined as up to 10 years or more than 10 years. Is there a rationale for this coding? Also, elsewhere in the text 15 years was mentioned. How does this fit the coding? [Also described on p.18 of the MS]. | In the introduction section (p.4), we noted that Cullen et al. identified that several evidence items cited in industry submissions about minimum unit pricing had been published more than 15 years before the consultation. To reduce the potential for confusion, we have replaced the mention of 15 years with “a decade” (line 76-77, p.4):  Several evidence items cited in the industry submissions were published more than *a decade* before the consultation…  Cullen et al. established the importance of considering the timeliness of evidence; however, Cullen et al. did not identify a reasonable timeframe for evidence that we could adopt in our study.  While there is no direct source for the time since publication criterion in the present study (up to 10 years or more than 10 years), we believe that within 10 years of the consultation reflects a reasonable timeframe that would be expected to capture a substantial volume of evidence that is also likely to be relevant to the contemporary advertising environment. |
| 12. p. 19 MS pdf. Line 276 ‘Where detected, analyses were re-run with extreme scores removed to determine whether results differed.’ Q. isn’t it stated later that there were no cases of extreme scores? | Thank you for identifying this opportunity for us to improve clarity regarding potential outlier scores.  To improve the consistency in language referring to outlier effects across the methods and results sections, we have amended the statistical analysis section as follows (line 276-277, p.13):  Data were eyeballed for *potential outlier* scores. Where detected, analyses were re-run with *outlier* scores removed to determine whether results differed.  We have amended the results section as follows (line 339, p.18):  *Potential outlier scores were identified.* *When analyses were re-run with outlier scores removed,* no outlier effects were detected. |
| 13. p. 27 of MS pdf. Principle findings brings together the most significant findings of the research. The authors give a very good succinct summation of the main findings. | Thank you for this positive feedback. |
| 14. Final para. ‘Modifications to consultation processes, such as exclusion of industry actors, quality requirements for submitted evidence, minimum standards for referencing evidence, and requirements to declare potential conflicts, may improve the transparency and application of evidence and public health outcomes of policy consultations’. These are very reasonable recommendations that come out of this work with application more broadly to government inquiries. If submissions draw on evidence that is no publicly traceable for inspection/accuracy/bias, then this is a shortcoming that could seemingly be risk managed along lines suggested. | Thank you for this positive feedback. |
| 15. Under acknowledgments, this reviewer is not sure of the relevance of the declarations that presumably apply to different authors’ contributions to submissions that would have been included in the inquiry. Acknowledgments usually refer to funding sources and the sort of statements made here should perhaps go in potential limitations and how/why bias is/not introduced and also discuss whether the reported research in this article was designed after or contemporaneously with these organisational submissions in which authors were involved. Alternatively, some of this may belong in declarations of interest. Since authors have contributed to some of the submissions to the inquires under analysis and in one case, to the final report. This does not mean to say the person should not be an author but to explain why these contributions do/not bias the research. | We thank Reviewer 1 for considering the appropriate placement of our declarations. We have revised our competing interests statement as noted at point 1 above. |
| **Reviewer 2** |  |
| 16. Many thanks for the opportunity to review this well written and important manuscript. The authors have conducted a thorough analysis of responses to two public consultations focusing on alcohol advertising policy using previously published methods. In this way the study is an important contribution to the growing body of literature on industry use and representation of evidence in the consultation process and adds a novel dimension by analysing and comparing industry with non-industry submissions. | We appreciate this positive feedback. |
| 17. Page 5 – paragraph from lines 91-103 – the authors could extend this further by making reference to additional studies that show that similar tactics are employed by the ultra-process food industry and the sugar sweetend beverage industry e.g.   Fooks GJ, Williams S, Box G, Sacks G. Corporations’ use and misuse of evidence to influence health policy: a case study of sugar-sweetened beverage taxation. Globalization and Health 2019; 15(1): 56.  Lauber K, McGee D, Gilmore AB. Commercial use of evidence in public health policy: a critical assessment of food industry submissions to global-level consultations on non-communicable disease prevention. BMJ Global Health 2021; 6(8): e006176.  This could help to further demonstrate the cross-industry nature of these tactics. | Thank you for these suggestions and particularly for suggesting Lauber et al. (2021), which was published after our manuscript was submitted to PLOS One.  We have amended the paragraph indicated by the reviewer to make reference to the studies of evidence cited by food and sweetened beverage industry actors (line 103-104, p.5):  *Evidence cited in submissions by ultra-processed food companies has similarly been found to lack independence from industry and other indicators of quality [Lauber et al., 2021]. Researchers have recognised parallels in evidence-related tactics by different corporate sectors [Fooks et al., 2019; Knai et al., 2021; Lauber et al., 2021] and* noted the potential value of applying *similar analyses* to other public health policy areas where corporate interests seek to influence policy, such as alcohol.  Fooks et al. (2019), Lauber et al. (2021), and Knai et al. (2021) were added to the reference list:  *Knai C, Petticrew M, Capewell S, Cassidy R, Collin J, Cummins S, et al. The case for developing a cohesive systems approach to research across unhealthy commodity industries. BMJ Glob Health. 2021;6(2).*  We have also integrated Fooks et al. (2019) and Lauber et al. (2021) references into the discussion section, as per point 20. |
| 18. Page 8 lines 167-172 – the authors describe who was classified as industry – where their other commercial actors e.g. those from the tourism/entertainment industry or bars/clubs (where these considered “retailers”) and where these included as industry or non-industry? This may be helpful in thinking though some of the wider partnership and allies that can be built by the industry and whose interests are served or threatened by different policy outcomes as the authors have shown for the advertising industry. | Supplementary Table 1 provides a breakdown of the submitters classified as either industry or non-industry actors. There were no submissions from licensed businesses such as bars/clubs or the tourism/entertainment industry to either consultation (unless they were among the three unidentified confidential submissions that were not publicly released). |
| 19. Page 22: “In both studies, significantly lower proportions of evidence cited by industry actors were highly relevant and featured indicators of higher quality compared to the points of comparison (i.e., systematic review evidence in Hatchard et al. and evidence cited by non-industry actors in the present study)” The authors may want to add some addition text to clarify that industry submissions have lower levels of features indicating high quality. | It is noted in several places in the results and discussion sections that evidence cited by industry actors was less likely to feature indicators of higher quality (e.g., lines 356-357 and 397-398). We have not included additional text to avoid repetition. |
| 20. Discussion/conclusion – the authors make several well-considered recommendations on how the consultation process may be strengthened. They may wish to highlight to the reader that similar recommendations have been made in other studies in the context of other policies and industries, which may strengthen the case for calling for such adjustments given other research groups in other locations and time points have also demonstrated the need for such measures if policy-making is to serve the public interest and draw on the best available evidence. | Thank you for this suggestion.  We have added the reference below to the sentence, “Policy consultations may benefit from the introduction of quality requirements for submitted evidence…” (line 417-418, p.23):  *Lauber K, McGee D, Gilmore AB. Commercial use of evidence in public health policy: a critical assessment of food industry submissions to global-level consultations on non-communicable disease prevention. BMJ Global Health 2021; 6(8): e006176.*  We have added two references to the sentence, “While the age of evidence can be readily assessed by policymakers, assessing independence from vested interests may be more difficult…” (line 419-420, p.23) and added Grundy et al. (2020) to the reference list:  *Grundy Q, Dunn AG, Bero L. Improving researchers' conflict of interest declarations. BMJ. 2020;368:m422.*  *Lauber K, McGee D, Gilmore AB. Commercial use of evidence in public health policy: a critical assessment of food industry submissions to global-level consultations on non-communicable disease prevention. BMJ Global Health 2021; 6(8): e006176.*  We have added the references below to the sentence, “To further aid the assessment of independence in policy consultations, it may be useful for the administering agency to define potential conflicts of interests and require submitters to declare any potential conflicts relevant to the evidence they present” (line 423-426, p.23):  *Evans-Reeves KA, Hatchard JL, Gilmore AB. ‘It will harm business and increase illicit trade’: an evaluation of the relevance, quality and transparency of evidence submitted by transnational tobacco companies to the UK consultation on standardised packaging 2012. Tobacco Control. 2015;24:e168–e77.*  *Hatchard JL, Fooks GJ, Evans-Reeves KA, Ulucanlar S, Gilmore AB. A critical evaluation of the volume, relevance and quality of evidence submitted by the tobacco industry to oppose standardised packaging of tobacco products. BMJ Open. 2014;4:e003757.*  *Lauber K, McGee D, Gilmore AB. Commercial use of evidence in public health policy: a critical assessment of food industry submissions to global-level consultations on non-communicable disease prevention. BMJ Global Health 2021; 6(8): e006176.*  We have acknowledged the similar experiences of Fooks et al. (2019) and Lauber et al. (2021) in the referencing practices of submitters obstructing the identification and appraisal of evidence (line 457-459, p.25):  Second, *consistent with difficulties experienced by those examining industry submissions to consultations in other policy contexts (e.g., ultra-processed foods and sugar-sweetened beverages [Fooks et al., 2019; Lauber et al., 2021])*, the referencing practices within submissions impacted the researchers’ ability to identify cited evidence. These practices varied between submissions as there was no requirement to reference evidence in a manner that would allow others to locate it or even to reference evidence at all. |
